# A comprehensive genetic and phylogenetic study of *Trypanosoma* spp. in bats and sand flies from shared habitats in Thailand

**DOI:** 10.1186/s13071-025-06934-5

**Published:** 2025-07-26

**Authors:** Samiullah Soomro, Siwaporn Tuangpermsub, Thongchai Ngamprasertwong, Morakot Kaewthamasorn

**Affiliations:** 1 The International Graduate Program of Veterinary Science and Technology (VST), Faculty of Veterinary Science, Chulalongkorn University, Bangkok, 10330 Thailand; 2 Center of Excellence in Veterinary Parasitology, Department of Pathology, Faculty of Veterinary Science, Chulalongkorn University, Bangkok, 10330 Thailand; 3 Veterinary Pathobiology Graduate Program, Faculty of Veterinary Science, Chulalongkorn University, Bangkok, 10330 Thailand; 4 Department of Biology, Faculty of Science, Chulalongkorn University, Bangkok, 10330 Thailand

**Keywords:** Phlebotomine sand fly, Bat, Trypanosome, Phylogenetics, Species delimitation, Pairwise genetic-distances, Thailand

## Abstract

**Background:**

Bats are known reservoirs for various pathogens, many of which can infect other animals through blood-feeding arthropods. Over 100 bat species have been identified as hosts for kinetoplastid protozoans, including ≥ 30 distinct *Trypanosoma* spp. However, bat trypanosomes remain relatively understudied owing to the nocturnal behavior of their hosts and legal restrictions on their capture for research. In Southeast Asia, particularly Thailand, only one study has investigated bat trypanosomes, leaving their distribution and transmission pathways largely unexplored.

**Methods:**

Between April 2021 and November 2023, bats were captured at ten locations across four provinces in Thailand. Blood samples were collected, examined microscopically, and screened for *Trypanosoma* DNA targeting the *SSU rRNA* and *gGAPDH* genes. Phlebotomine sand flies from bat sampling sites were collected and analyzed for *Trypanosoma* DNA and blood meal sources. Sequences were identified using BLASTn searches, while genetic relationships were assessed through pairwise genetic distance, phylogenetic reconstruction, and TCS haplotype network analyses. In addition, species delimitation was conducted to validate unidentified sequences at the species level.

**Results:**

Out of 368 bats, 40 (10.9%) tested positive for four *Trypanosoma* species (including two previously named: *T. dionisii* and *T. noyesi*). Out of 189 sand flies, a single one tested positive for an unnamed anuran trypanosome from a gravid female (*Phlebotomus stantoni*), and the study was unable to detect the blood source of this sand fly. In total, 37 pools (189 specimens) of female sand flies—comprising 159 unfed, 29 gravid, and 1 engorged specimen—were analyzed for vertebrate blood meals, but none tested positive. Multiple analyses (BLASTn, phylogenetics, haplotype networks, pairwise genetic distances, and species delimitation) also confirmed a *Trypanosoma* sp. in a gravid sand fly, along with *T. dionisii* and *T. noyesi*, plus two uncharacterized bat-associated species.

**Conclusions:**

The *Trypanosoma* spp. detected in the present study aligns with prior reports of diverse trypanosomes in bat populations, reinforcing their role as key reservoirs. Notably, a single sand fly (*Phlebotomus stantoni*) tested positive for an unnamed anuran trypanosome, but its blood meal source could not be determined, leaving unresolved questions about potential transmission pathways.

**Graphical abstract:**

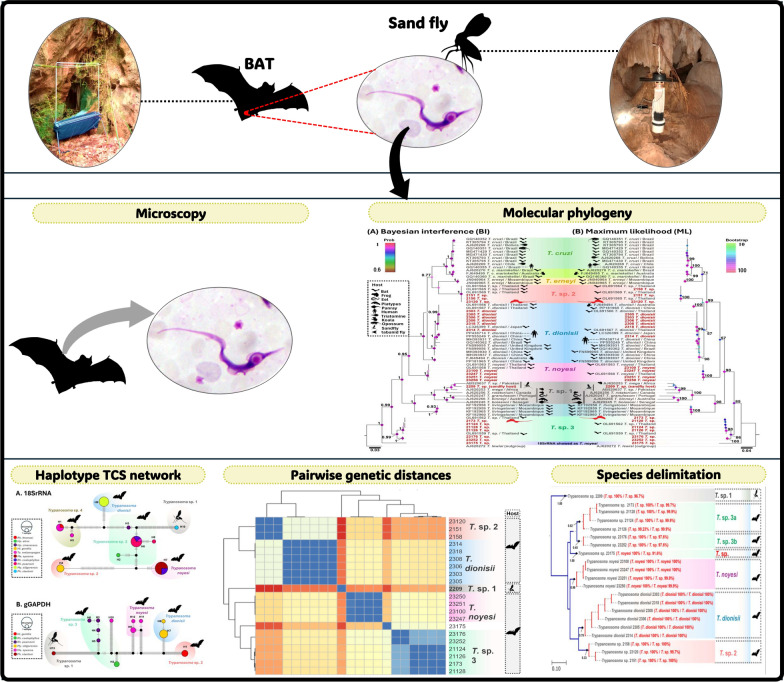

**Supplementary Information:**

The online version contains supplementary material available at 10.1186/s13071-025-06934-5.

## Background

Bats represent a highly diverse group of mammals, and are the only mammals capable of sustained flight, with some species migrating long distances from their roosting sites. As reservoir hosts for a wide range of infectious pathogens, bats harbor viruses such as rabies and coronaviruses [1, 2], as well as vector-borne bacteria and parasites, including *Bartonella*, *Rickettsia*, *Borrelia* [3, 4], *Babesia*, *Leishmania*, and *Trypanosoma* [5–8]. The potential for migrating bats in the geographic spread of these pathogens is a growing concern. Among the *Trypanosoma* spp. documented in bats, the subgenus *Schizotrypanum* is commonly identified across South America, Europe, Africa, and Asia [6, 9–11]. More than 100 bat species carry ≥ 30 *Trypanosoma* spp., including *T*. *dionisii*, *T*. *cruzi*, *T*. *vespertilionis*, and *T*. *rangeli* [12, 13].

Despite these findings, research on bat trypanosomes remains limited, primarily due to the logistic and regulatory challenges. As nocturnal and legally protected wildlife, bats cannot be studied without special permits from regulatory authorities in many countries. The genus *Trypanosoma* comprises approximately 500 species of flagellated protozoans, which are dixenous (requiring two hosts), infect vertebrates—including humans—and are transmitted by hematophagous invertebrates such as leeches, tsetse flies, ticks, and triatomine bugs [14, 15]. These parasites infect a variety of vertebrate classes, including mammals, with some species posing significant risks to human and animal health [16–20]. While tsetse flies are vectors of African trypanosomes, they are not representative of trypanosome vectors globally and are not indigenous to Asia, suggesting that regional transmission may occur via other hematophagous arthropods, such as mosquitoes and black flies. Sand flies are also suspected of transmitting trypanosomes to snakes, lizards, frogs, and bats. In southern Thailand, the first evidence of *Trypanosoma* sp. DNA in the sand fly, *Phlebotomus stantoni*, has been reported [21–26]. However, more studies on the role of sand flies in trypanosome transmission in the region remain lacking.

Phlebotomus is a well-known vector of *Leishmania*, while several sand fly species, including *Sergentomyia gemmea*, *Se*. *barraudi*, *Se*. *iyengari*, *Se*. *khawi*, and *Grassomyia indica*, have been implicated in carrying *Leishmania* [27–31]. In addition, *Se*. *khawi*, *Se*. *barraudi*, *Gr*. *indica*, *Ph*. *asperulus*, and *Ph*. *stantoni* have been implicated in carrying *Trypanosoma* sp. [25, 29, 31, 32], underscoring the possible involvement of sand flies in trypanosome transmission. Although the DNA of *Leishmania* has been found in *Sergentomyia gemmea* and *Sergentomyia barraudi*, it is still unclear whether they serve as effective vectors, largely owing to insufficient understanding of their biology as vectors [33, 34]. Blood meal analysis of phlebotomine sand flies using the *COI* gene has identified a range of vertebrate hosts, including birds, frogs, sun skinks, and bats. Although *T*. *noyesi* is not classified within the subgenus *Schizotrypanum*, but phylogenetically belongs to the *T. cruzi* clade—which traces its origins to ancestral bat trypanosomes—recently had its DNA detected in the sand fly species *Idiophlebotomus longiforceps* in Thailand [35–37]. This finding reinforces the possible role of sand flies in the transmission of bat trypanosomes. In a previous investigation, *T*. *noyesi* was isolated from *Megaderma spasma* bats residing in caves in Thailand [38], which shared a genetic lineage with *T*. *noyesi* detected in *Ph*. *asperulus* sand flies. This discovery supports the hypothesis that a bat-associated *Trypanosoma* sp. is closely linked to sand flies inhabiting cave environments [31].

Although no confirmed cases of *T. noyesi* infection have been reported in humans, it is vital to recognize that certain trypanosome spp. within the *T. cruzi* group may pose potential public health risks [36]. *T*. *dionisii*, once considered nonpathogenic, proliferates in mammalian cells in vitro and forms amastigotes in the skeletal muscle of *Pipistrellus pipistrellus* bats [39, 40]. Although historically regarded as bat-specific, *T*. *dionisii* has been detected in the cardiac tissues of a 2-year-old Brazilian child with acute Chagas disease (caused by *T*. *cruzi*), presumably acquired through oral transmission [41]. Recent reports of human infections in China highlight its zoonotic potential, particularly as genetic analyses have revealed close similarities between human-derived strains and those from *Eptesicus serotinus* bats in China [42]. Collectively, these findings suggest that *T*. *dionisii* is not restricted to animal hosts and may represent an emerging zoonotic threat. Given its widespread distribution, further research on bat trypanosomes is essential to better understand their potential impact on public health.

To date, research on bat trypanosomes in the region has been limited, with only a single study investigating their presence in Thailand [38]. Moreover, the absence of an integrated approach—encompassing both bat and sand fly sampling—has hindered a comprehensive understanding of host–vector interactions in trypanosome transmission. This study aimed to simultaneously screen trypanosomes in diverse bat hosts and sand flies and conduct blood meal analysis.

## Methods

### Study locations and sample collections

**Fig. 1:**
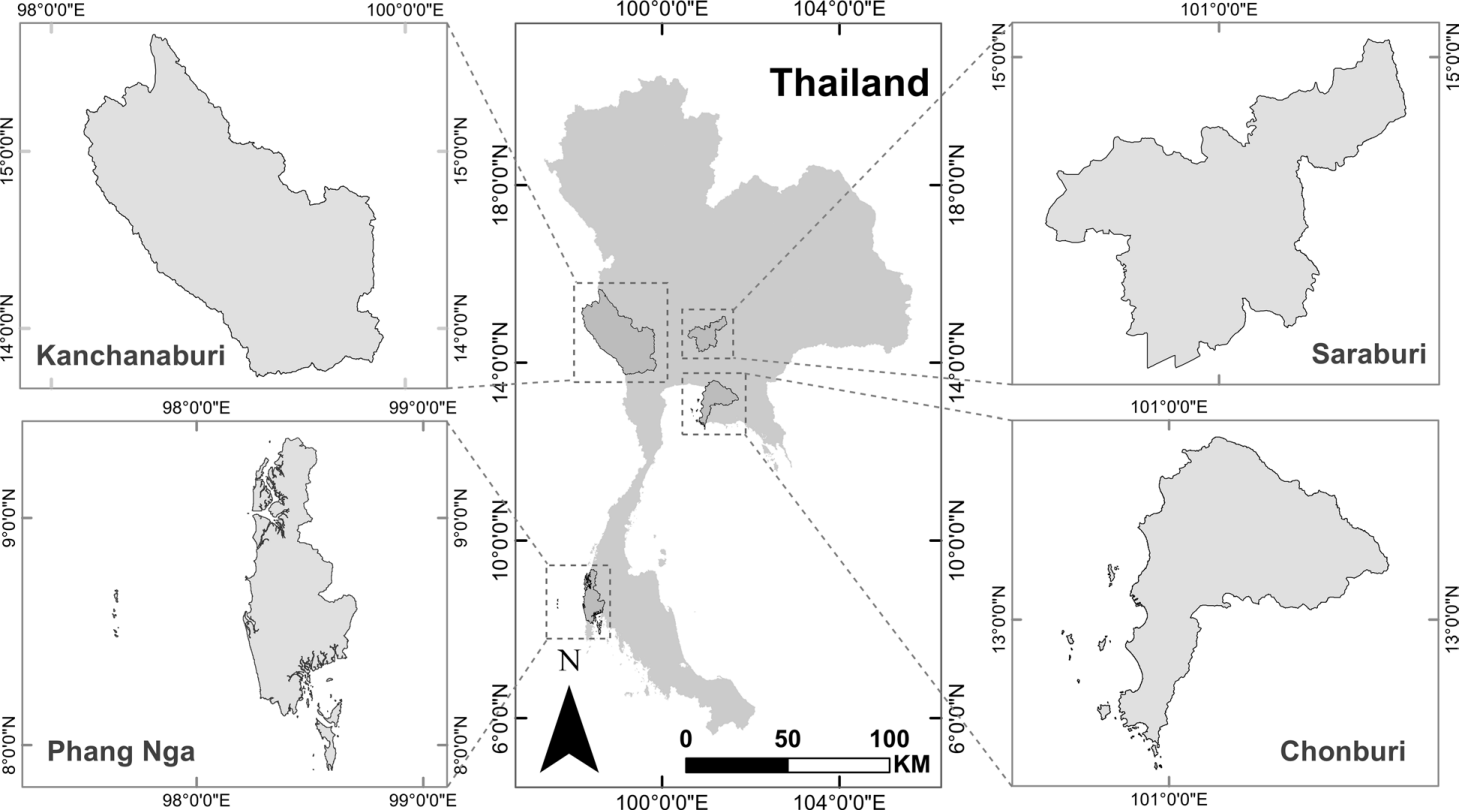
Geographic map depicting the nine sampling locations across four provinces in Thailand, where bat blood and sand fly samples were collected (see Table 1 for details). The map was generated using ArcGIS version 10.7.1 (https://enterprise.arcgis.com/) and includes a scale bar (in km) to illustrate the distances between sampling sites

Between April 2021 and November 2023, bats were captured as part of ongoing surveys on their pathogens [3, 38, 43, 44] from ten sampling sites, including caves, islands, and wildlife locations across four provinces in Thailand (Fig. 1; Table 1). Blood samples were collected, while individuals weighing < 2.5 g were excluded to ensure their safety. Female phlebotomine sand flies were captured as previously described [44]. Sampling sites were selected using a convenience sampling approach in collaboration with the local officials from the Department of National Parks, Wildlife, and Plant Conservation. Bats were captured and identified using methodologies adapted from Arnuphapprasert et al. [43] and Francis [45]. When necessary, echolocation calls were analyzed to confirm species identification. In addition, bats were categorized by sex and reproductive status. Blood samples were collected following the protocol described in a previous study [43]. Immediately after sample collection, all bats were released at their respective capture sites. The samples were transported to the Faculty of Veterinary Science at the Chulalongkorn University, Bangkok, Thailand, for further analysis.

**Table 1:** Bat collection and prevalence of trypanosomes in various locations of four provinces in Thailand

Province	Location	Latitude	Longitude	No. bat captured	No. blood sampled	No. positive (%)
Phang Nga	**KYI**	08° 07′ 25.0″ N	98° 36′ 22.0″ E	31	31	1 (2.5)
	TR	08° 03′ 17.5″ N	98° 35′ 49.4″ E	2	2	0 (0)
Kanchanaburi	**PHC**	14° 24′ 37.6″ N	98° 51′ 13.4″ E	285	209	21 (52.5)
	PF	14° 24′ 36.3″ N	98° 51′ 15.2″ E	1	1	0 (0)
	**MGC**	14° 21′ 29.8″ N	98° 56′ 11.0″ E	16	16	3 (7.5)
	**DC**	14° 27′ 59.0" N	98° 49′ 51.0" E	11	11	**7** (17.5)
	TKC	14° 20′ 32.5″ N	98° 57′ 28.3″ E	9	9	0 (0)
	**MPC**	14° 21′ 19.6″ N	98° 56′ 14.1″ E	89	75	**7** (17.5)
Saraburi	KCP	14° 31′ 29.0" N	101° 02′ 06" E	2	0	0 (0)
Chonburi	**KKI**	12° 34′ 19.0" N	100° 55′ 59.8" E	14	14	1 (2.5)
	Total			460	368	40 (100)

The letter codes listed below signify the sampling locations of bats: Koh Yao Noi Island (KYI), Thiwson Resort (TR), Phra Cave (PHC), Pomelo Farm (PF), Ma Glue Cave (MGC), Darwvading Cave (DC), Taklor Cave (TKC), Manow Phee Cave (MPC), Kaeng Khoi-Cham Phak Phaeo (KCP), and Koh Kham Island (KKI). Sampling locations that are highlighted in bold signify trypanosome positivity

### Microscopic examination

Thin capillary blood films were prepared on-site and stained following the protocol outlined by Riana et al. [38]. Subsequently, slides were examined microscopically using an ECLIPSE Si microscope equipped with a DS-Fi3 camera and NIS-Elements L imaging software (Nikon Corporation, Tokyo, Japan).

### DNA extraction

Genomic DNA (gDNA) was extracted from the blood samples with the NucleoSpin^®^ Blood Kit (Macherey–Nagel, Düren, Germany) following the manufacturer’s instructions. To concentrate the DNA, the final elution volume was reduced to 25 µL, as recommended in a previous study [43]. For sand fly specimens, gDNA was extracted from the whole bodies using the NucleoSpin^®^ Tissue Kit (Macherey–Nagel), following the manufacturer’s protocol, but with a modified elution step. Specifically, to enhance DNA yield, the volume of the preheated BE buffer (70 °C) was reduced from 100 to 60 µL before column elution. The extracted gDNA was stored at −20 °C until further analysis.

### Amplification of Trypanosoma DNA in bat samples

Nested and semi-nested polymerase chain reactions (PCRs) were employed to amplify the small subunit ribosomal RNA (*SSU rRNA*) and glycosomal glyceraldehyde-3-phosphate dehydrogenase (*gGAPDH*) genes using reference primers (Supplementary Table S1). The thermal cycling conditions for each gene were adjusted per the respective protocols of each primer set (Supplementary Table S2). For *SSU rRNA*, the initial PCR was performed in a total volume of 12.5 µL using the primers TRY927F and TRY927R [46, 47]. The reaction mixture comprised 1.75 µL of sterile distilled water, 0.375 µL of both forward and reverse primers (final concentration: 0.3 μM), 2.5 µL of dNTPs, 6.25 µL of 2× KOD Fx Neo Buffer, 0.25 µL of KOD Fx Neo polymerase (Toyobo, Japan), and 1 µL of gDNA. The secondary PCR utilized the primers SSU561F and SSU561R [48] and 2 µL of the first-round PCR product as the template. The reaction mix included 0.25 µL each of the forward and reverse primers (final concentration: 0.25 μM), 1 µL of dNTPs (0.2 μM), 1.25 µL of 10× Ex Taq buffer, 0.125 µL (0.3125 U) of Ex Taq polymerase (TaKaRa Bio, USA), and 7.625 µL of the first-round PCR product diluted 1:5 in sterile distilled water. PCR amplification was performed on either the MiniAmp^™^ Thermal Cycler (Applied Biosystems^™^, MA, USA) or the Axygen^®^ MaxyGene II Thermal Cycler (Corning, CA, USA). For amplifying *gGAPDH*, the first-round PCR used KOD Fx Neo (Toyobo) with the primers GAPDHF and GAPDHR [49] in a total reaction volume of 12.5 µL. For the second round, the primers GAPDHF and g4a [50] were employed. A 1:5 dilution of the first-round PCR product in sterile water served as the template for the second round PCR. It employed the Ex Taq polymerase (TaKaRa Bio) in a final reaction volume of 12.5 µL. For both *SSU rRNA* and *gGAPDH*, *T*. *cruzi* strain G gDNA was utilized as a positive control, while sterile distilled water was the negative control. The second-round PCR products were electrophoresed on a 1.5% agarose gel stained with ethidium bromide and visualized under a UV transilluminator. For samples yielding positive results, the PCR reaction volume was increased from 12.5 µL to 50 µL to ensure sufficient DNA for sequencing, followed by electrophoresis under the same conditions. To purify the PCR products, the ExoSAP-IT^™^ Kit (Applied Biosystems, Lithuania) was utilized to remove excess primers and nucleotides if nonspecific bands were absent. If present, the PCR products were purified via agarose gel extraction employing the NucleoSpin^®^ Gel and PCR Clean-up Kit (Macherey–Nagel) following the manufacturer’s instructions. The purified PCR products were eluted in 25 µL of buffer, and their concentration and quality were assessed using a NanoDrop spectrophotometer (Thermo Scientific). Finally, the purified PCR products were bidirectionally sequenced by the Sanger method at the U2Bio Co., Ltd. (https://www.u2bio.co.th/home), a commercial sequencing service provider.

### Detection of trypanosome DNA in sand fly samples

Trypanosome DNA was amplified using KOD FX Neo polymerase (Toyobo) and the primers TRY927F and TRY927R [46, 47]. Conventional PCR was performed in a total reaction volume of 12.5 µL to amplify the *SSU rRNA* segment (900 bp) from the gDNA isolated from sand fly samples. As previously described, *gGAPDH* was amplified using a semi-nested PCR approach, with the primers GAPDHF–GAPDHR [49] in the first-round and GAPDHF–g4a [50] in the second round. In the second round, 2 µL of the undiluted first-round PCR product was used as the template, rather than the diluted one. Details of the primers and PCR conditions are provided in Supplementary Tables S1 and S2. For samples that yielded positive electrophoresis results, the PCR reaction volume was increased from 12.5 µL to 50 µL to ensure sufficient DNA for sequencing. Electrophoresis and sequencing were then repeated using the same protocols.

### Identification of host bloodmeal DNA in sand fly samples

Sand flies were categorized into three physiological states: unfed, gravid, or engorged. The cytochrome c oxidase subunit I (*COI*) gene from vertebrates was amplified using DNA extracted from sand flies to identify the origins of blood meals. Specimens were pooled by species on the basis of previous taxonomic confirmation [44]. The number of specimens per pool ranged from one to eight, depending on specimen abundance and availability. For species represented by more than eight specimens, four to six individuals of the same species were pooled per sample randomly, but ensuring the inclusion of at least one or two gravid specimens per pool when available. A specific primer set, VertCOI_7194_F and Mod_RepCOI_R [51], was utilized in PCR to generate amplicons ~395 bp long. This primer pair was designed specifically for vertebrate DNA amplification, following the methodology described by Reeves et al. [51]. PCR was performed in a total volume of 12.5 µL, comprising 0.75 µL of sterile distilled water, 0.375 µL each of the forward and reverse primers (final concentration: 0.3 μM), 2.5 µL of dNTPs, 6.25 µL of 2× KOD Fx Neo Buffer, 0.25 µL of KOD Fx Neo polymerase (Toyobo), and 2 µL of genomic DNA. Further details regarding the primers and PCR conditions are provided in Supplementary Tables S1 and S2.

### Nucleotide sequence editing and BLASTn similarity search

Nucleotide sequences were carefully examined, manually refined, and trimmed to generate consensus sequences using BioEdit version 7.2.5 [52] for further analysis. Haplotype analysis was conducted employing DnaSP version 6.12.03 [53]. The processed *SSU rRNA* and *gGAPDH* sequences were then queried against the GenBank^™^ database using BLASTn to assess query coverage and percentage identity (Supplementary Table S3).

### Phylogenetic analyses and species delimitations

Study and reference sequences were analyzed using Bayesian inference (BI) and maximum likelihood (ML) methods (Supplementary Tables S4 and S5). BI analysis used MrBayes on XSEDE through CIPRES (https://www.phylo.org), with a 25% burn-in and 10,000,000 Markov chain Monte Carlo (MCMC) iterations. The best-fitting evolutionary models for BI analysis were determined utilizing MODELTEST in MEGA 11 [54], based on the lowest Bayesian information criterion (BIC) scores. Phylogenetic trees were visualized using FigTree v1.4.4 (http://tree.bio.ed.ac.uk/software/figtree). ML analysis utilized IQ-TREE (http://iqtree.cibiv.univie.ac.at/), with 1000 bootstrap replicates, and the optimal model was selected automatically. The Poisson tree processes (PTP) model was used to delineate *Trypanosoma* spp. by applying the bPTP web server (https://species.h-its.org/), as outlined in Zhang et al. [55]. In addition, a ML phylogenetic analysis of the concatenated *SSU rRNA* and *gGAPDH* sequences employed default parameters outlined previously [56].

### TCS haplotype networks and pairwise distance matrix

The MEGA 11 software was used to concatenate nucleotide sequences [54], and the TCS haplotype network was constructed using PopART software, version 1.7 [57]. In RStudio version 2024.09.1 + 394 (https://posit.co/download/rstudio-desktop/), the necessary R packages (*readxl*, *pheatmap*, and *ape*) were installed to generate a heatmap, and pairwise genetic distances between *SSU rRNA* and *gGAPDH* sequences were analyzed. A heatmap illustrating the genetic distance matrix was generated with the *pheatmap* package.

## Results

### Bat collection

A total of 460 bats were captured, and blood samples were collected from 368 individuals (Supplementary Table S6), representing seven families: Craseonycteridae (*n* = 21, 5.7%), Emballonuridae (*n* = 63, 17.1%), Hipposideridae (*n* = 153, 41.6%), Megadermatidae (*n* = 22, 6.0%), Miniopteridae (*n* = 1, 0.3%), Rhinolophidae (*n* = 44, 12.0%), and Vespertilionidae (*n* = 64, 17.4%), encompassing 17 species. The bat species with the maximal number of blood samples was *Hipposideros gentilis* (*n* = 120, 32.6%), followed by *Taphozous melanopogon* (*n* = 63, 17.2%) and *Myotis siligorensis* (*n* = 61, 16.6%). Species from which only a single sample was collected included *Hipposideros armiger*, *Hipposideros larvatus*, *Miniopterus magnater*, *Rhinolophus refulgens*, and *Myotis ricketti*. In terms of sampling locations, a majority of the blood samples were obtained from the Phra Cave (PC; *n* = 209, 56.8%), followed by the Manow Phee Cave (MPC; *n* = 75, 20.4%) and the Koh Yao Noi Island (KYI; *n* = 31, 8.5%). The least number of samples was collected at Kaeng Khoi-Cham Phak Phaeo (KCP; *n* = 0, 0.0%), followed by Pomelo Farm (PF; *n* = 1, 0.3%) and the Thiwson Resort (TR; *n* = 2, 0.5%) (Tables 1 and 2).

**Table 2:** Detection of trypanosomes in seven bat families from nine sites (including caves and islands) across four provinces in Thailand

Family	Bat species	Location (no. blood sampled)	Total no. blood sampled	Blood film +ve	No. sequences obtained		Prevalence in % ^1^
					*SSU rRNA*	*gGAPDH*	
Craseonycteridae	*Craseonycteris thonglongyai*	PHC (21)	21	0	0	0	0 (0/21)
Emballonuridae	***Taphozous melanopogon***	PHC (49), KKI (14)	63	0	5	0	7.9 (5/63)
Hipposideridae	***Hipposideros atrox***	KYI (29)	29	0	1	0	3.4 (1/29)
	***Hipposideros gentilis***	PHC (114), MPC (6)	120	2	4	3	3.3 (4/120)
	***Hipposideros cineraceus***	PHC (2)	2	0	1	0	50 (1/2)
	*Hipposideros armiger*	DC (1)	1	0	0	0	0 (0/1)
	*Hipposideros larvatus*	MPC (1)	1	0	0	0	0 (0/1)
Megadermatidae	***Megaderma spasma***	PHC (11), TKC (8), DC (3)	22	0	9	5	40.9 (9/22)
Miniopteridae	*Miniopterus magnater*	PHC (1)	1	0	0	0	0 (0/1)
Rhinolophidae	*Rhinolophus refulgens*	KYI (1)	1	0	0	0	0 (0/1)
	*Rhinolophus malayanus*	KYI (1), MPC (3)	4	0	0	0	0 (0/4)
	***Rhinolophus pearsonii***	PHC (2), DC (7)	9	1	7	4	77.8 (7/9)
	***Rhinolophus coelophyllus***	PHC (6), MGC (10), MPC (10), TKC (1)	27	0	5	2	18.5 (5/27)
	***Rhinolophus thomasi***	PHC (3)	3	0	1	0	33.3 (1/3)
Vespertilionidae	*Myotis muricola*	TR (2)	2	0	0	0	0 (0/2)
	***Myotis siligorensis***	MGC (6), MPC (55)	61	0	7	6	11.5 (7/61)
	*Myotis ricketti*	PF (1)	1	0	0	0	0 (0/1)
	Total		368	3	40	20	10.9 (40/368)

^1^Number of infected individuals/total individuals are given in parenthesis. The letter codes listed below signify the sampling locations of bats: Phra Cave (PHC), Manow Phee Cave (MPC), Koh Kham Island (KKI), Koh Yao Noi Island (KYI), Darwvading Cave (DC), Taklor Cave (TKC), Thiwson Resort (TR), Ma Glue Cave (MGC), and Pomelo Farm (PF). Bat species that are highlighted in bold signify trypanosome positivity

### Prevalence of trypanosomes in bats

Of 368 bats, 40 (10.9%) tested positive for *Trypanosoma* spp., on the basis of at least one positive PCR result of *SSU rRNA* or *gGAPDH*, or through microscopic examination. The highest number of *Trypanosoma*-positive samples was detected at the Phra Cave (PHC; 21/209, 10.1%), followed by the Darwvading Cave (DC; 7/11, 63.7%) and the Manow Phee Cave (MPC; 7/75, 9.4%). In contrast, the lowest number of infections was observed at the Ma Glue Cave (MGC; 3/16, 18.8%), the Koh Yao Noi Island (KYI; 1/31, 3.3%), and the Koh Kham Island (KKI; 1/14, 7.2%). No *Trypanosoma* infections were detected at the Thiwson Resort (TR), the Pomelo Farm (PF), or Tham Khao Chong (TKC). Among the bat families examined, Rhinolophidae contributed the greatest number of infected individuals (13/44, 29.5%), followed by Megadermatidae (9/22, 41.0%), and Vespertilionidae (7/64, 10.9%). At the species level, the maximal infection prevalence was observed in *Rhinolophus pearsonii* (7/9, 77.8%), followed by *Hipposideros cineraceus* (1/2, 50.0%), *Megaderma spasma* (9/22, 40.9%), and *Rhinolophus thomasi* (1/3, 33.3%). Conversely, the lowest was recorded in *Hipposideros gentilis* (4/120, 3.3%), *Hipposideros atrox* (1/29, 3.4%), *Taphozous melanopogon* (5/63, 7.9%), *Myotis siligorensis* (7/61, 11.5%), and *Rhinolophus coelophyllus* (5/27, 18.5%). No *Trypanosoma* infections were detected in eight bat species: *Craseonycteris thonglongyai* (*n* = 21), *Hipposideros armiger* (*n* = 1), *H*. *larvatus* (*n* = 1), *Miniopterus magnater* (*n* = 1), *Rhinolophus refulgens* (*n* = 1), *R*. *malayanus* (*n* = 4), *Myotis muricola* (*n* = 2), and *M*. *ricketti* (*n* = 1) (Tables 1 and 2).

### Molecular detection of *Trypanosoma* DNA in sand flies

In total, 189 female sand fly specimens, representing 14 species from 13 sites across four provinces in Thailand, were examined for *Trypanosoma* DNA by PCR amplification of *SSU rRNA* and *gGAPDH* genes. Among the specimens analyzed, only a single sand fly, identified as *Ph*. *stantoni* collected from the entrance of the Tiger Cave (TGC) in Saraburi Province, tested positive for *Trypanosoma*. This specimen exhibited a successful amplification of the *SSU rRNA* (895 bp) and *gGAPDH* (763 bp) genes, resulting in an overall *Trypanosoma* infection rate of 0.5% (1/189) in phlebotomine sand flies (Table 3). No *Trypanosoma* DNA was detected in sand fly specimens collected from the remaining 12 locations in Kanchanaburi, Chachoengsao, and Phatthalung provinces (Table 3), suggesting a low prevalence of trypanosomes in sand flies in these regions. However, detecting *Trypanosoma* DNA only in a single specimen does not confirm the vectorial competency of sand flies, as no attempts were made to culture and observe parasite development from this specimen.

**Table 3:** Molecular detection of trypanosomes from 12 confirmed and 2 putative novel species of sand fly collected across 13 locations in four provinces of Thailand

Province	Location	PCR (*SSU rRNA* and *gGAPDH*)		No. of obtained sequences	
		No. tested	No. positive	*SSU rRNA* (895 bp)	*gGAPDH* (763 bp)
Saraburi	TGC^a^	9	1	1	1
	CUC^e^	6	0	0	0
	CUC^d^	22	0	0	0
Kanchanaburi	PHCL^a^	23	0	0	0
	PHCL^b^	41	0	0	0
	MGC^a^	11	0	0	0
	MGC^c^	18	0	0	0
	MPC^b^	20	0	0	0
	TKC^b^	11	0	0	0
	TKC^a^	2	0	0	0
	PHCS^b^	14	0	0	0
Chachoengsao	MRF^f^	10	0	0	0
Phatthalung	PWHR^g^	2	0	0	0
Total		189	1	1	1

The following letter codes represent the sampling locations used in this study: PHCLa: Phra Cave (large), PHCLb: Phra Cave (large), PHCSb: Phra Cave (small), TKCb: Taklor Cave, TKCa: Taklor Cave, TGCa: Tiger Cave, MGCa: Ma Glue Cave, MGCc: Ma Glue Cave, MPCb: Manow Phee Cave, CUCd: Chulalongkorn University Campus, CUCe: Chulalongkorn University Campus, MRFf: Murrha Farm, PWHRg: Phatthalung Wildlife Husbandry Research Station. The following labels refer to specific sampling areas: a = cave entrance, b = inside cave, c = cave underground, d = forest, e = banana farm, f = buffalo farm, g = wildlife sanctuary. Detection was conducted using conventional PCR targeting *SSU rRNA* and semi-nested PCR targeting *gGAPDH*

### Identification of blood meal source in sand flies

In total, 37 pools (189 specimens) of female sand flies, representing 14 species, were examined for vertebrate blood meal analysis using the mitochondrial *COI* gene. Among them, 159 (84.1%) were classified as unfed, 29 (15.3%) as gravid, and 1 (0.5%) as engorged. The most commonly identified species were *Phlebotomus barguesae* (72 individuals, 38.1%), followed by *Sergentomyia anodontis* (33 individuals, 17.5%) and *Sergentomyia* spp. 1 (17 individuals, 9.0%). The least frequent were *Sergentomyia siamensis* and *Grassomyia indica*, each represented by a single individual (0.5%). A majority of the collected specimens were gravid females, with 11 individuals of *Ph*. *barguesae* and 9 for *Se*. *anodontis*. However, only one engorged female, *Ph. stantoni*, was detected. A total of 37 pools, comprising 159 unfed specimens, 29 gravid specimens, and 1 engorged specimen, were analyzed; however, none tested positive for vertebrate blood meals (Table 4).

**Table 4:** Blood meal analysis of 14 phlebotomine sand fly species based on the vertebrate *COI* gene

Species	Unfed	Gravid	Engorged	Total	Total of pools	Positive pools
*Phlebotomus barguesae*	61	11	0	72	12	0
*Phlebotomus stantoni*	3	3	1	7	1	0
*Idiophlebotomus asperulus*	11	1	0	12	2	0
*Idiophlebotomus longiforceps*	8	0	0	8	1	0
*Sergentomyia anodontis*	24	9	0	33	6	0
*Sergentomyia sylvatica*	14	0	0	14	3	0
*Sergentomyia perturbans*	2	0	0	2	1	0
*Sergentomyia barraudi*	5	1	0	6	1	0
*Sergentomyia hivernus*	1	0	0	1	1	0
*Sergentomyia khawi*	12	2	0	14	3	0
*Sergentomyia siamensis*	1	0	0	1	1	0
*Sergentomyia* sp. 1	15	2	0	17	3	0
*Sergentomyia* sp. 2	1	0	0	1	1	0
*Grassomyia indica*	1	0	0	1	1	0
Total	159	29	1	189	37	0

### *SSU rRNA* and *gGAPDH* phylogeny of trypanosomes from bats and sand flies

**Fig. 2:**
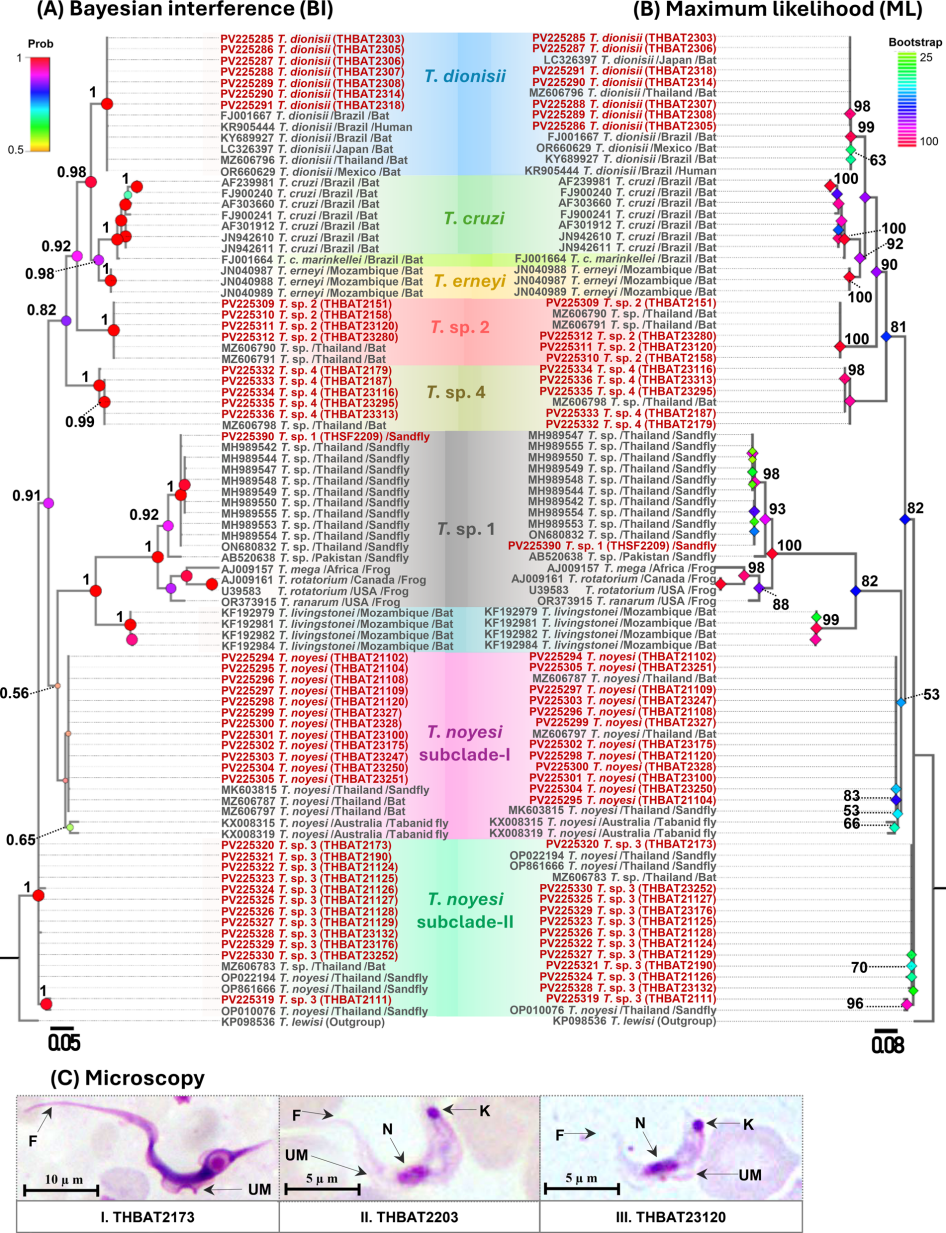
**A** Bayesian Inference (BI) and **B** maximum likelihood (ML) phylogenetic trees constructed using *SSU rRNA* sequences (524–560 bp) of trypanosomes obtained in this study, alongside reference sequences. ML bootstrap values > 50 and BI posterior probabilities > 0.5 are displayed at the nodes. Sequences obtained in this study are highlighted in red. The host and country of origin are indicated after the species names. **C** Microscopic images of the trypomastigote stages: (I) THBAT2173 from *Rhinolophus pearsonii*, (II) THBAT2203 from *Hipposideros gentilis*, and (III) THBAT23120 from *Hipposideros gentilis*. N, nucleus; K, kinetoplast; F, flagellum; UM, undulating membrane

**Fig. 3:**
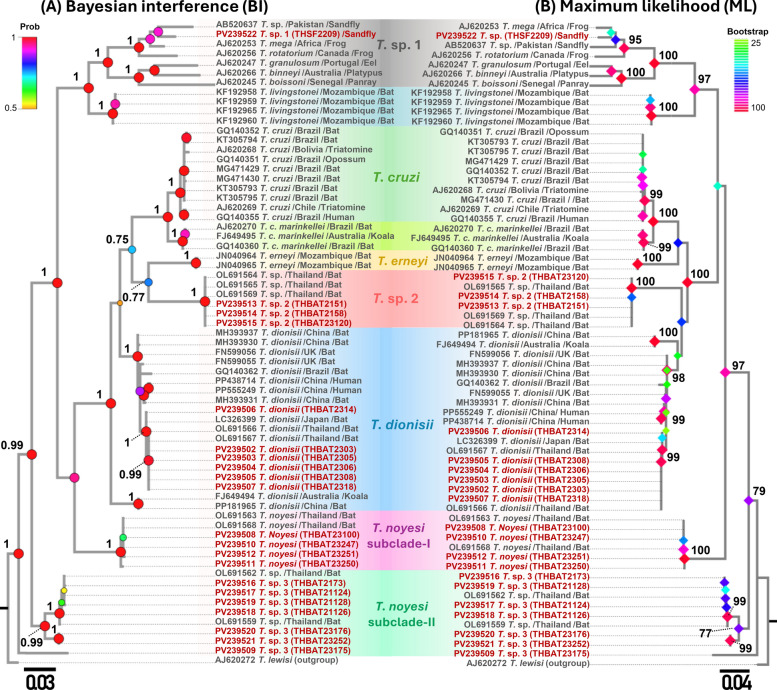
**A** Bayesian Inference (BI) and **B** maximum likelihood (ML) phylogenetic trees based on *gGAPDH* sequences (753 bp) of trypanosomes identified in this study, alongside reference sequences. ML bootstrap values > 50 and BI posterior probabilities > 0.5 are provided at the nodes. Sequences obtained in this study are shown in red. The host and country of origin are indicated after the species names

A total of 40 trypanosome *SSU rRNA* sequences (524–560 bp) from bats and 1 from a sand fly, along with 20 *gGAPDH* sequences (763 bp) from bats and 1 from a sand fly, were obtained and classified into six distinct clades (Figs. 2 and 3). *SSU rRNA* analysis revealed that seven *T*. *dionisii* isolates from bats clustered with global sequences from Brazil, Japan, Mexico, and Thailand, formed a monophyletic clade. This clade included a human-derived Brazilian sequence (KR905444), supported by high statistical values (BI = 1, ML = 99) (Fig. 2a-b). Similarly, *gGAPDH* analysis revealed that six bat-derived isolates grouped with reference strains from Australia, Brazil, China, Japan, Thailand, and the UK, confirming broad distribution. The analysis also linked *T*. *dionisii* to two human isolates (PP438714 and PP555249) from China (Fig. 3a-b).

The *T*. *noyesi* clade included 12 bat *SSU rRNA* sequences, clustering with reference sequences from bats, sand flies, and tabanid flies in Thailand and Australia. For *gGAPDH*, four sequences grouped with Thai bat *T*. *noyesi*, while one (PV239509) formed a distinct basal clade. Phylogenetic analyses (BI = 1, ML = 100) placed *Trypanosoma* sp. 1 in a distinct clade with anuran trypanosomes. The *SSU rRNA* sequence from a sand fly clustered with isolates from sand flies and frogs across Canada, Pakistan, Thailand, and the USA. *gGAPDH* analysis revealed association with diverse hosts (sand flies, frogs, eels, platypuses, panrays) spanning Africa, Canada, Portugal, Australia, Senegal, and Pakistan, demonstrating wide host range and geographic distribution.

Both *SSU rRNA* and *gGAPDH* analyses robustly supported (BI = 1, ML = 100) the placement of bat-derived *Trypanosoma* sp. 2 with an unidentified Thai bat *Trypanosoma* sp.—four isolates by *SSU rRNA* and three by *gGAPDH*. The phylogenetic analysis of bat *Trypanosoma* sp. 3 using *SSU rRNA* showed that 12 isolates clustered with unidentified *Trypanosoma* sp. from bat and *T*. *noyesi* from sand flies in Thailand. While *gGAPDH* analysis grouped seven *Trypanosoma* sp. 3 isolates with an unidentified *Trypanosoma* sp. from a Thai bat. *Trypanosoma* sp. 3 likely represents a sister lineage to *T*. *noyesi*, although this is not fully supported by the topology of the phylogenetic tree. Phylogenetic analysis of *Trypanosoma* sp. 4 from bats was conducted using *SSU rRNA* and *gGAPDH* genes, though data availability varied between the two markers. Five *Trypanosoma* sp. 4 isolates clustered with an unidentified Thai bat *Trypanosoma* sp. in *SSU rRNA*, but amplification with *gGAPDH* was unsuccessful. In addition, the concatenated ML phylogenetic tree, constructed on the basis of *SSU rRNA* and *gGAPDH* genes (provided in Supplementary Fig. S1), supported the results of the individual phylogenetic trees for both genes.

### Microscopy of the trypanosomes

According to microscopic examination, three blood films were positive for trypanosomes from *Rhinolophus pearsonii* (THBAT2173, PCR positive) and *Hipposideros gentilis* (THBAT2203, PCR negative; THBAT23120, PCR positive). Trypomastigotes in *Hipposideros gentilis* exhibited a consistent C- or U-shaped morphology with a visible nucleus, kinetoplast, flagellum, and undulating membrane (Fig. 2c). In contrast, the nucleus and kinetoplast were not observed in trypomastigotes from *Rhinolophus pearsonii*.

### TCS haplotype network

The TCS haplotype network was constructed using trypanosome *SSU rRNA* and *gGAPDH* sequences, obtained from nine bat and one sand fly species. An analysis of 41 *SSU rRNA* genes identified 10 genetic variants, while 21 *gGAPDH* genes revealed 13 genetic variants (details including host species are mentioned in Supplementary Fig. S2) (Fig. 3).

### Comparative pairwise distances of trypanosome *SSU rRNA* and *gGAPDH* genes

**Fig. 4:**
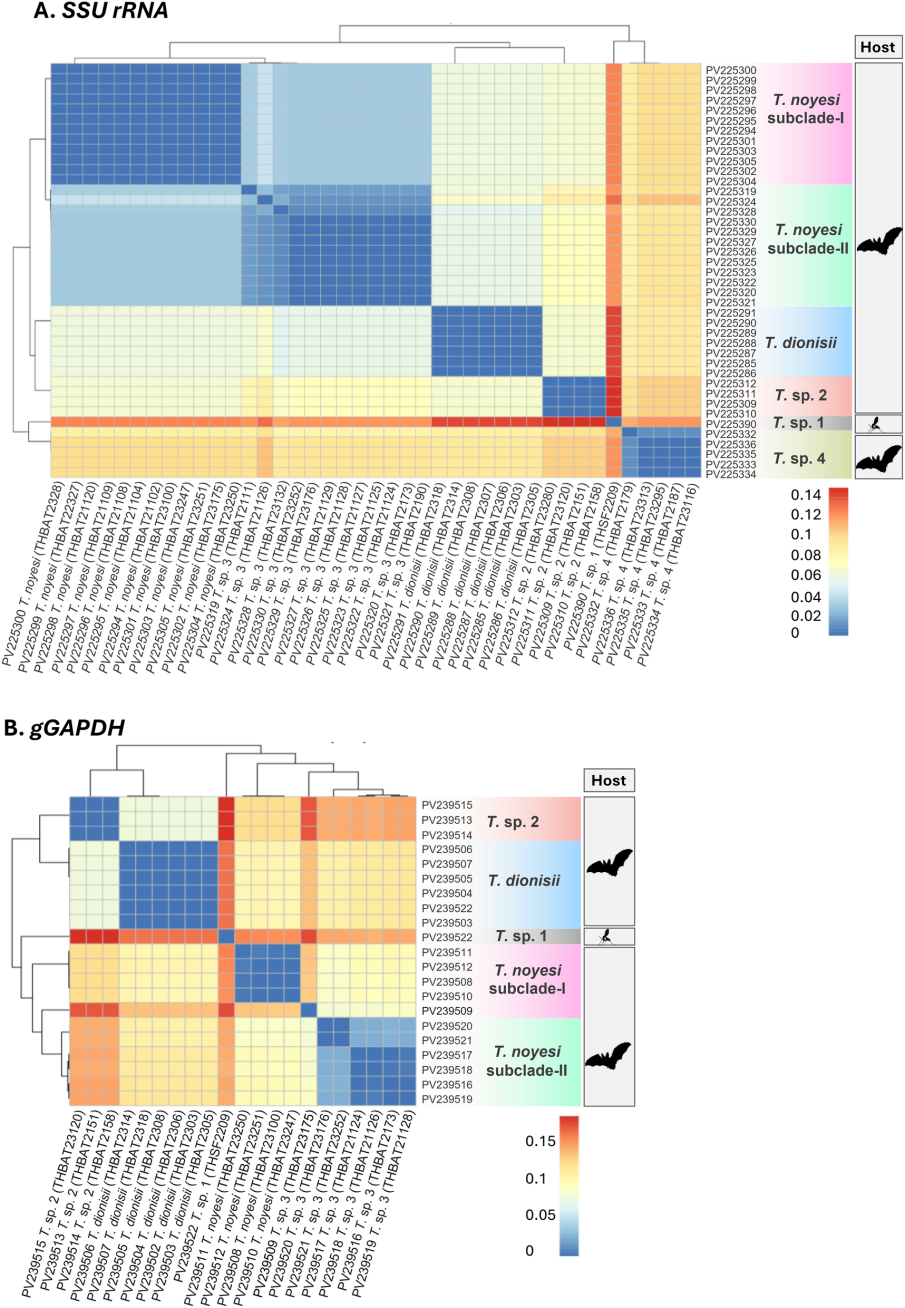
A heatmap of the pairwise genetic distances calculated using the Kimura 2-parameter model (K80) for **A** *SSU rRNA* (524–560 bp) and **B** *gGAPDH* (753 bp) sequences obtained from bats and phlebotomine sand flies in this study. Color gradients, represented by the scale bars on the right-hand side of each panel, indicate pairwise genetic distances

Concerning *SSU rRNA* and *gGAPDH*, most clades exhibited low intraspecific genetic distances (≤ 0.01), indicating a high degree of intraspecific genetic similarity. In contrast, interspecific genetic distances ranged from 0.03 to 0.15 for *SSU rRNA* and from 0.09 to 0.19 for *gGAPDH*, with clustering patterns revealing evident genetic divergence between clades. Across both genetic markers, the *T*. *dionisii*, *T*. *noyesi* (*T. noyesi* subclade-I and II), *T*. sp. 1, *T*. sp. 2, and *T*. sp. 4 clades exhibited minimal intraspecific genetic variation (≤ 0.01), reinforcing a strong genetic similarity level among sequences within each clade. Moreover, *T*. *noyesi* exhibits minimal interspecific genetic variation (0.03–0.04 for *SSU rRNA* and 0.08–0.10 for *gGAPDH*), with *T*. sp. 3, strongly supporting that *T*. sp. 3 likely represents a sister lineage to *T*. *noyesi* (Fig. 4). Notably, the *T*. sp. 1 clade, derived from sand flies, exhibited substantial interspecific genetic diversity (ranging from 0.11 to 0.15 for *SSU rRNA* and from 0.14 to 0.19 for *gGAPDH*) when compared with bat-associated *Trypanosoma* lineages, suggesting the emergence of a distinct evolutionary descent (Fig. 4).

### Putative species delimitation of *Trypanosoma* spp.

**Fig. 5:**
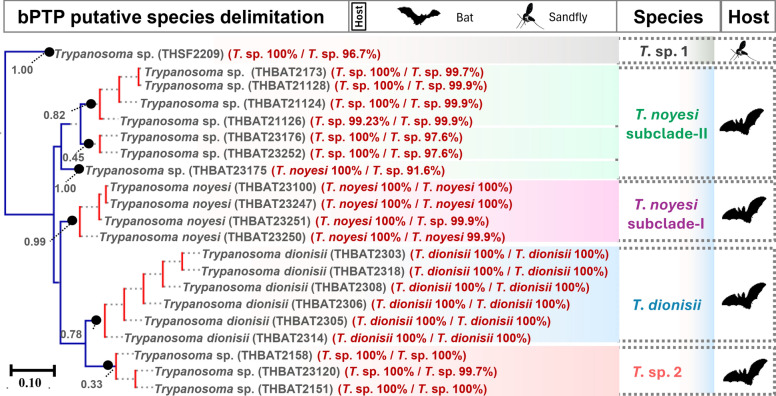
Putative species delimitation of *Trypanosoma* spp. on the basis of concatenated *SSU rRNA* (524–560 bp) and *gGAPDH* (753 bp) sequences (total length: 1277–1313 bp). Monophyletic groups in red indicate single putative species, while terminal branches in blue represent distinct lineages. Numbers at the terminal ends of species names correspond to the sample IDs of the host species

Species delimitation identified seven putative *Trypanosoma* spp. from bats and phlebotomine sand flies in Thailand (Fig. 5). The sand fly *Trypanosoma* isolate formed a distinct lineage (*T*. sp. 1) with strong Bayesian support (1.00), positioned peripherally relative to the bat-associated *Trypanosoma* clades. Such analysis further recognized four, six, and three isolates of *T*. *noyesi*, *T*. *dionisii*, and *T*. sp. 2 as separate species, respectively. Notably, six isolates of *T*. sp. 3 were classified into two putative species (*T*. sp. 3a and 3b). Interestingly, a single isolate (THBAT23175) was identified as a separate putative species on the basis of bPTP analysis, despite exhibiting complete genetic similarity (0.00 genetic distance) with *T*. *noyesi* based on *SSU rRNA*. Due to a failed *gGAPDH* amplification, the *T*. sp. 4 *SSU rRNA* sequences were excluded from species delimitation.

## Discussion

In this study, the overall trypanosome infection rate in bats was 10.9% (40/368), which exceeds previously reported rates in Asia, including Thailand (6.6%) [38]; Yunnan, China (6.17%) [48]; Shandong, China (10.3%) [11]; and Japan (2.13%) [10]. This higher prevalence may indicate more intense transmission dynamics within the study area. Moreover, while previous research identified ten bat species as *Trypanosoma* hosts in Thailand [38], our study expands this list by documenting *Hipposideros atrox* and *H*. *cineraceus* as additional hosts, suggesting a broader host range than previously recognized. Notably, most bat and sand fly samples collected in this study were from the Kanchanaburi province, which may introduce sampling bias; therefore, the results should be interpreted cautiously. Notably, only one prior study in Thailand had documented *Trypanosoma* spp. in bats, specifically reporting *T*. *dionisii* in *Myotis siligorensis* from the Ma Glue Cave (Cave D) in Kanchanaburi and the Chomphon Cave in Ratchaburi [38]. In contrast, our study presents the first detection of *T*. *dionisii* in *My*. *siligorensis* from the Manow Phee Cave in Kanchanaburi. In addition, we identified an unidentified *Trypanosoma* (*T*. sp. 1) in sand fly, raising the possibility of them contributing to trypanosome transmission. However, since this study did not attempt to culture the parasite from sand fly salivary glands or feces, nor did it screen other hematophagous insects in the region, the role of sand flies as vectors remains to be investigated in detail. Furthermore, given the low trypanosome infection rate in sand flies, it is unlikely that they serve as primary biological vectors for bat trypanosomes.

Moreover, we were unable to detect the host blood source, even in gravid and engorged females, likely because gravid sand flies had already fully digested their blood meals to support egg development. This likely explains the absence of detectable vertebrate DNA. These findings are consistent with those of previous studies by Abbasi et al. [58], Kent [59], and Oshaghi et al. [60], which reported similar challenges in identifying blood meals in hematophagous insects.

In China, *T*. *dionisii* has been detected in bats at a higher rate using *gGAPDH* than *SSU rRNA* [11], while in this study, *SSU rRNA* yielded a higher detection rate than *gGAPDH*. These differences in detection efficiency may be attributable to variations in primer specificity, amplification sensitivity, and regional genetic diversity [11]. The phylogenetic analyses grouped the studied isolates within the *T*. *dionisii* clade, together with strains from bats in Australia, Brazil, China, Japan, Mexico, UK, and Thailand [10, 38, 61–66], confirming their genetic relatedness to globally distributed variants and broad geographic distribution consistent with prior research [9, 10, 13, 58, 61, 67]. This study found that *T*. *dionisii* isolates from Thailand grouped into a monophyletic clade with a Brazilian human cardiac strain (KR905444) on the basis of *SSU rRNA* analysis, while *gGAPDH* analysis showed clustering with two human isolates from China (PP438714, PP555249) [41, 42]. These findings support its evolutionary links to human-infecting strains.

Phylogenetic analyses positioned *T*. *noyesi* isolates within the *T*. *noyesi* clade, alongside global ones derived from bats, sand flies, and tabanid flies in Thailand and Australia [31, 37, 38]. This clustering suggests that bats may serve as reservoirs, while sand and tabanid flies act as vectors, facilitating the transmission of *T*. *noyesi* across various ecosystems [26, 32, 35]. In addition, our study expands the known host range by detecting *T*. *noyesi* in *M*. *spasma* and *Rhinolophus coelophyllus* bats, indicating more complex transmission dynamics. Interestingly, an isolate (THBAT23175) of *T*. *noyesi*, with 91.6% identity, formed a distinct clade based on TCS haplotype network, phylogenetic, pairwise genetic distance, and species delimitation analyses for *gGAPDH*.

*T*. *noyesi* and an unidentified *Trypanosoma* sp. have been previously detected in various sand fly species across multiple provinces in Thailand, including Chiang Rai, Trang, Phatthalung, and Songkhla [25, 26, 32, 35, 68]. To our knowledge, this is the first study to integrate simultaneous collection of sand flies and bats from shared habitats for *Trypanosoma* screening. Notably, we report the first detection of an unidentified *Trypanosoma* sp. in *Ph*. *stantoni* from Tiger Cave (Saraburi province), though we were unable to capture bats at this site. Phylogenetic, haplotype network, genetic distance, and species delimitation analyses confirmed *Trypanosoma* sp. 1 from sand flies as a distinct clade separate from bat-associated *Trypanosoma* lineages. In a previous study, *T*. *noyesi* was documented in bats in Thailand, revealing a shared genetic lineage with *T*. *noyesi* from the *Ph*. *asperulus* sand fly [35]. Therefore, further investigations from the region are necessary to determine whether sand flies serve as competent vectors for bat trypanosomes.

In addition to *T*. *dionisii* and *T*. *noyesi*, we identified three previously unrecognized *Trypanosoma* spp. (*T*. sp. 2,* T*. sp. 3, and *T*. sp. 4) in bats from Thailand, highlighting greater trypanosome diversity than previously documented. Phylogenetic analysis, TCS haplotype networks, pairwise genetic distances, and species delimitation techniques robustly supported the identification of *T*. sp. 2 as a distinct clade, signifying potential host specificity within *Hipposideros gentilis* [38]. In this study, *Trypanosoma* sp. 3 sequences from bats revealed 97.6–100% identity with an unidentified *Trypanosoma* sp. from Thailand [38]. Interestingly, on the basis of *SSU rRNA* gene analysis, three *T*. *noyesi* isolates (OP022194, OP010076, and OP861666) were sourced from sand flies clustered within the *T*. sp. 3 clade, intimating possible misidentifications in previous studies conducted in Thailand [35, 68]. This strongly indicates the need for further taxonomic reassessment utilizing additional genetic markers or morphological characteristics. Moreover, *T*. sp. 3 exhibits minimal interspecific genetic variation with *T*. *noyesi*, strongly supporting that *T*. sp. 3 likely represents a sister lineage to *T*. *noyesi*. Finally, five *Trypanosoma* sp. 4 *SSU rRNA* sequences from bats exhibited 98.9–100% identity with an unidentified *Trypanosoma* sp. (MZ606798) from Thailand [38]. Unfortunately, we were unable to amplify the *gGAPDH* gene from these isolates, which might be attributed to either the sensitivity of the primers or the genetic variations present in the primer regions among different genes [11].

## Conclusions

The present study identifies a diverse assemblage of trypanosomes in bats and sand flies, including *T*. *dionisii*, *T*. *noyesi*, and three uncharacterized lineages. In addition, the detection of *T*. *noyesi* in multiple bat species, including newly recognized hosts, suggests complex host–vector interactions. However, further research is needed to assess the vectorial competence of sand flies for the transmission of bat trypanosomes.

## Supplementary Information

Additional file 1.

Additional file 2.

Additional file 3.

Additional file 4.

Additional file 5.

Additional file 6.

Additional file 7.

Additional file 8.

## Data Availability

The nucleotide sequences generated from this research have been submitted to the GenBank^™^ database (https://www.ncbi.nlm.nih.gov/nuccore) as detailed in Supplementary Table S4.

## Abbreviations

BLASTn: Basic local alignment search tool for nucleotides
BOLD: Barcode of Life Database
bPTP: Bayesian Poisson tree processes
*gGAPDH*: Glycosomal glyceraldehyde-3-phosphate dehydrogenase
MEGA: Molecular evolutionary genetics analysis
PCR: Polymerase chain reaction
*SSU rRNA*: Small subunit ribosomal ribonucleic acid
