# Supplementary material for: A comprehensive genetic and phylogenetic study of Trypanosoma spp. in bats and sand flies from shared habitats in Thailand
Source: Parasit Vectors. 2025 Jul 26;18:298. doi: 10.1186/s13071-025-06934-5 (PMC12297788; doi:10.1186/s13071-025-06934-5)
Supplement: Supplementary file 1 — Additional file 1. [file 13071_2025_6934_MOESM1_ESM.docx]

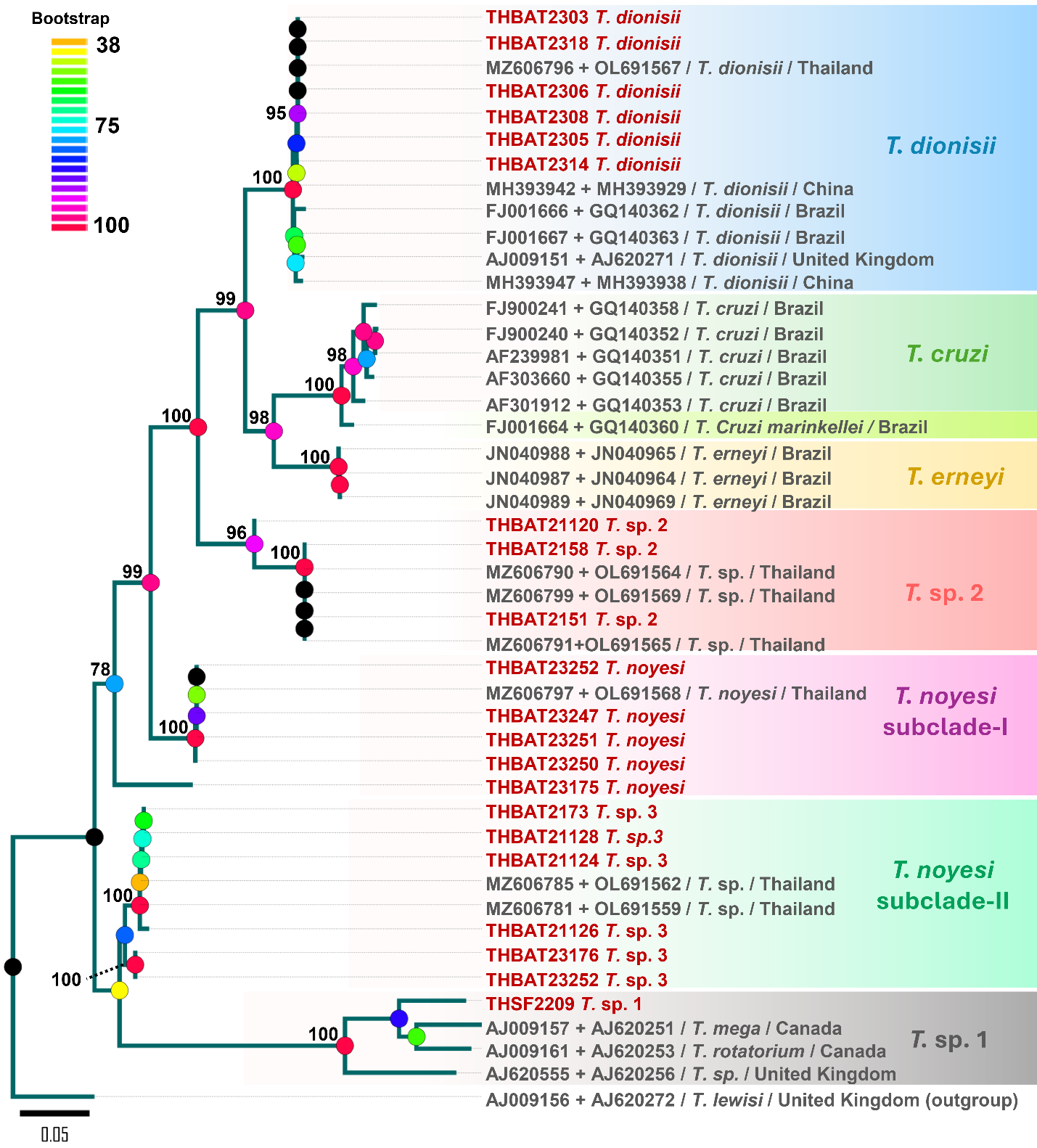


**Additional file 2: Supplementary Figure S1** Maximum Likelihood (ML) phylogenetic tree constructed using concatenated *SSU rRNA* (525-763bp) and *gGAPDH* (561-763) genes with a final dataset of 1288-1324 bp and 1,000 bootstrap replications. ML bootstrap values >38 are displayed with multicolored circles and numbers at the nodes. The accession numbers for the trypanosomes are indicated before Scientific names while country of origin are indicated after scientific names. The sequences obtained in this study are highlighted in red with specimen voucher ID before scientific name.
