# Supplementary material for: A comprehensive genetic and phylogenetic study of Trypanosoma spp. in bats and sand flies from shared habitats in Thailand
Source: Parasit Vectors. 2025 Jul 26;18:298. doi: 10.1186/s13071-025-06934-5 (PMC12297788; doi:10.1186/s13071-025-06934-5)
Supplement: Supplementary file 2 — Additional file 2. [file 13071_2025_6934_MOESM2_ESM.docx]

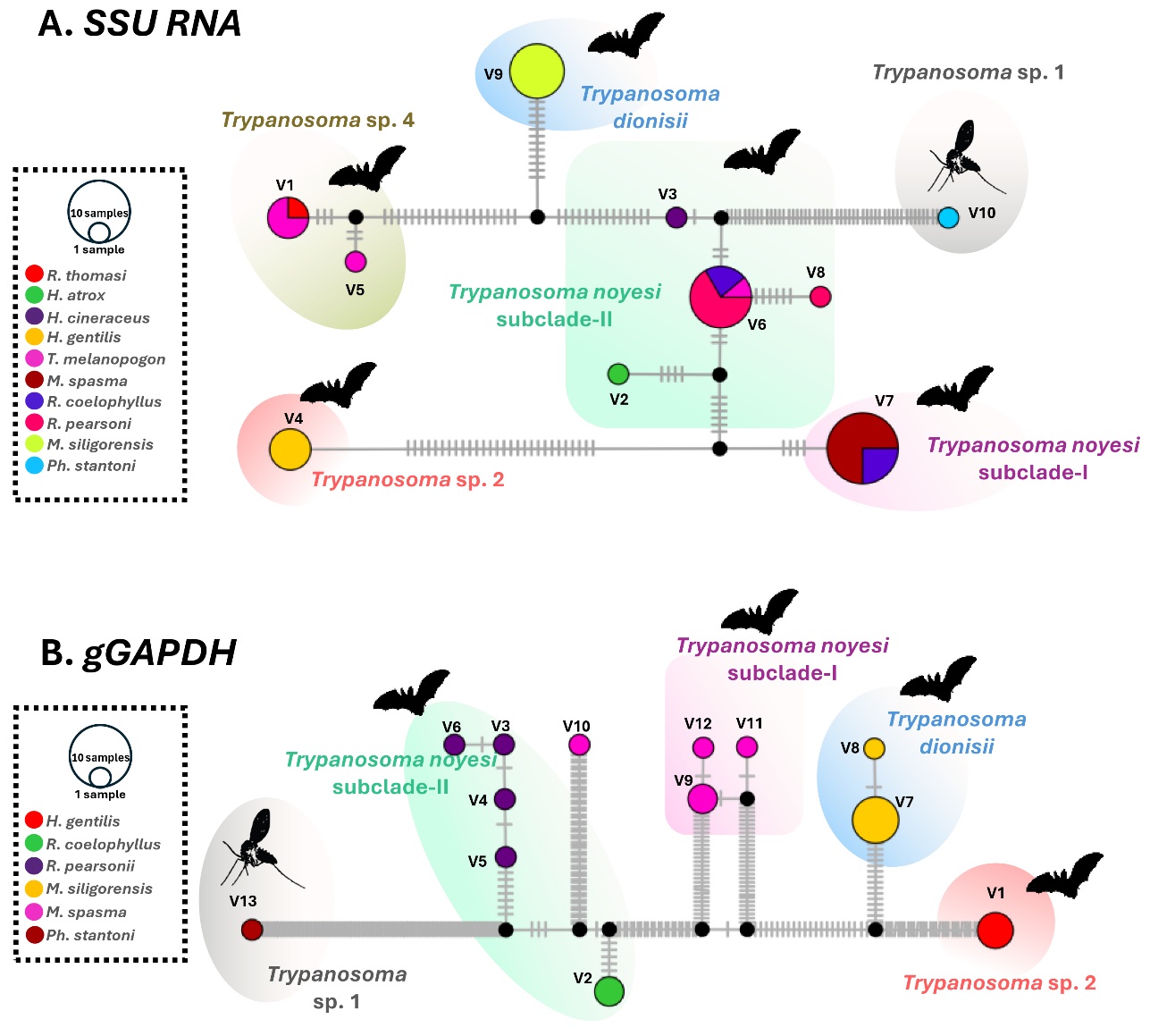


**Additional file 1: Supplementary Figure S2** TCS haplotype networks of *Trypanosoma* spp. detected in bats and sand flies based on **(A)** *SSU rRNA* (524–560 bp) and **(B)** *gGAPDH* (753 bp) sequences. Each genetic variant (V) is represented by a circle, with the circle size proportional to the number of individuals sharing that genetic variant. Lines connecting genetic variants represent nucleotide differences, indicating mutations from a common ancestral genetic variant. Missing genetic variants are indicated by black dots. Colors corresponding to host species (bats and sand flies) are annotated in the accompanying legend.

***TCS haplotype network***

The *SSU rRNA* genetic variants were classified into two identified species clusters—*T*. *dionisii* (one genetic variant) and *T*. *noyesi* (two genetic variants)—and four unidentified species clusters—*Trypanosoma* sp. 1 (one genetic variant), *T*. sp. 2 (one genetic variant), *T*. sp. 3 (four genetic variants), and *T*. sp. 4 (two genetic variants). Similarly, the *gGAPDH* genetic variants were assigned to two identified species clusters—*T*. *dionisii* (two genetic variants) and *T*. *noyesi* (three genetic variants)—along with three unidentified species clusters—*T*. sp. 1 (one genetic variant), *T*. sp. 2 (one genetic variant), and *T*. sp. 3 (six genetic variants).

For *T*. *dionisii*, all seven *SSU rRNA* isolates belonged to a single genetic variant (V9). In contrast, six *gGAPDH* isolates were classified into two distinct genetic variants (V7 and V8). Notably, all genetic variants of both genes were exclusively associated with a single bat species, *M*. *siligorensis*, collected from the Manow Phee Cave. In *T*. *noyesi*, all 12 *SSU rRNA* isolates were assigned to a single genetic variant (V7), which was linked to two bat species—*Megaderma spasma* and *Rhinolophus coelophyllus*—sampled from three caves (Phra, Ma Glue, and Daowadung). Conversely, among the five *gGAPDH* isolates, four were classified into three genetic variants (V9, V11, and V12), forming a distinct clade; one isolate (23175) was assigned to a separate clade representing genetic variant V10, associated with *Megaderma spasma* from the Phra Cave. For *T*. sp. 1, single *SSU rRNA* and *gGAPDH* genes were each assigned to a single genetic variant, V10 and V13, respectively, associated with the phlebotomine sand fly species *Ph. stantoni*, collected from the Tiger Cave. In *T*. sp. 2, four *SSU rRNA* and three *gGAPDH* isolates belonged to genetic variants V4 and V1, respectively, both linked to *Hipposideros gentilis* from the Phra Cave. In *T*. sp. 3, 12 *SSU rRNA* isolates were classified into four genetic variants: V2, V3, V6, and V8, associated with five bat species—*Hipposideros atrox*, *Hipposideros cineraceus*, *Rhinolophus pearsonii*, *Taphozous melanopogon*, and *Rhinolophus coelophyllus*—collected from Ko Yao Noi Island, Phra Cave, and Daowadung Cave. In contrast, seven *gGAPDH* isolates were assigned to five genetic variants: V2, V3, V4, V5, and V6, linked to *Rhinolophus pearsonii* and *R*. *coelophyllus* from the Phra and Daowadung Caves. For *T*. sp. 4, five *SSU rRNA* isolates were classified into two genetic variants (V1 and V5), associated with *Rhinolophus thomasi* and *Taphozous melanopogon* from the Phra Cave and Koh Kham Island, respectively. However, no *T*. sp. 4 isolates were detected for *gGAPDH*.
